# Dermoid Cyst of the Floor of the Mouth

**DOI:** 10.1100/tsw.2003.04

**Published:** 2003-03-24

**Authors:** Sergio M. Lima, Bruno R. Chrcanovic, Alfredo M.B. de Paula, Belini Freire-Maia, Leandro N. Souza

**Affiliations:** Universidade Federal de Minas Gerais, Belo Horizonte, Minas Gerais, Brazil

**Keywords:** dermoid cyst, floor of the mouth

## Abstract

Dermoid cysts of the floor of the mouth are rare lesions thought to be caused by entrapment of germinal epithelium during the closure of the mandibular and hyoid branchial arches. They usually present as a nonpainful swelling. This type of lesion occurs more frequently in patients between 15 and 35 years, but can be seen in all age ranges. Histologically, all dermoids are lined by epidermis. The contents of the cyst lining determine the histological categories of the cyst: epidermoid, if epidermis is lining the cyst; dermoid, if skin annexes exist; or teratoid, if there are tissues derivated from the three germinal layers. Anatomical classification is useful for surgical approach choice, intra- or extraorally. This report presents a case of a dermoid cyst of the floor of the mouth in a 12-year-old patient, and a review of all steps necessary for its diagnosis and treatment was made.

## INTRODUCTION

A dermoid cyst is a lesion thought to be caused by entrapment of germinal epithelium during the closure of the mandibular and hyoid branchial arches. These cysts are common in lines of fusion. Clinically, they usually present as a nonpainful swelling in the floor of the mouth, frequently of a doughy consistency, that may cause difficulty in eating, speaking[1,2], and breathing[3]. An ultrasound scan is commonly used as the first choice to investigate the lesion[4].

Floor of the mouth dermoid cysts have been identified in patients as young as newborns to those in their seventh decade of life. However, the highest incidence seems to occur in patients between 15 and 35 years, with a fairly equal distribution between males and females[3,5,6].

Histologically, all dermoids are lined by epidermis. The contents of the cyst lining determine the histological categories of the cyst[7]: epidermoid, if epidermis is lining the cyst; dermoid, if skin annexes exist; or teratoid, if there are tissues derivated from the three germinal layers.

Dermoid cysts are rare in the mouth. A review made in 1988[8] found that only 3 of 95 dermoid cysts of head and neck in children were in the floor of the mouth. In 1966[9], Taylor reported that only 6.5% of these cysts appears in the oral cavity. This report presents a case of dermoid cyst of the floor of the mouth in a 12-year-old patient, and a review of all steps necessary for diagnosis and treatment was made.

## CASE REPORT

Patient R.R.O., 12 years old, was referred to the Oral and Maxillofacial Surgery service, Hospital Maria Amélia Lins, Belo Horizonte, Minas Gerais complaining of a large submental mass. The history disclosed that the lesion had a slow and progressive growth. Inspection and palpation of the floor of the mouth and face revealed a smooth, doughy, lobular, compressible cystic mass measuring approximately 35 × 25 mm in the submental area (Fig. 1). Physical evaluation was unremarkable. His vital signs were stable and he was afebrile. The differential diagnosis was plunging ranula, dermoid cyst, benign pleomorphic adenoma, and cystic hygroma. Additional exams consisted of ultrasound imaging, radiographs, and routine laboratory tests. Routine laboratory findings were RBC 5.040.000/$mm^3$ and platelets 253.000/$mm^3$. Urinalysis, PT/PTT was normal. The patient was classified as ASA I.

Under general nasoendotracheal anesthesia, an incision was made around the perimeter of the mandible, at the submental area. The lesion was approached through a 4-cm extraoral, submental incision. After removal, the lesion was incised and found to be cystic, filled with thick white cheesy material (Fig. 2). The soft-tissue wound was closed with 4-0 polyglicolic sutures and skin with 6-0 mononylon. Prior to the procedure, 1 g of Cefalotin had been given intravenously.

Extubation was performed without complications. Normal tube feeing was initiated on the following day. No significant edema was observed and healing was uneventful and the patient was discharged on the second postoperative day. There was no recurrent 6 months after surgery.

Macroscopic examination showed a brownish sac-like soft tissue, with irregular shape and surface, measuring 30 × 20 × 18 mm. Histopathological aspects represent a simple form of cystic teratoma. The cyst is lined by a layer orthokeratinized squamous epithelium. The cyst lumen is generally filled with a mixture of desquamated keratin and sebum. The cyst capsule is composed of a narrow zone of compressed connective tissue exhibiting variable numbers of

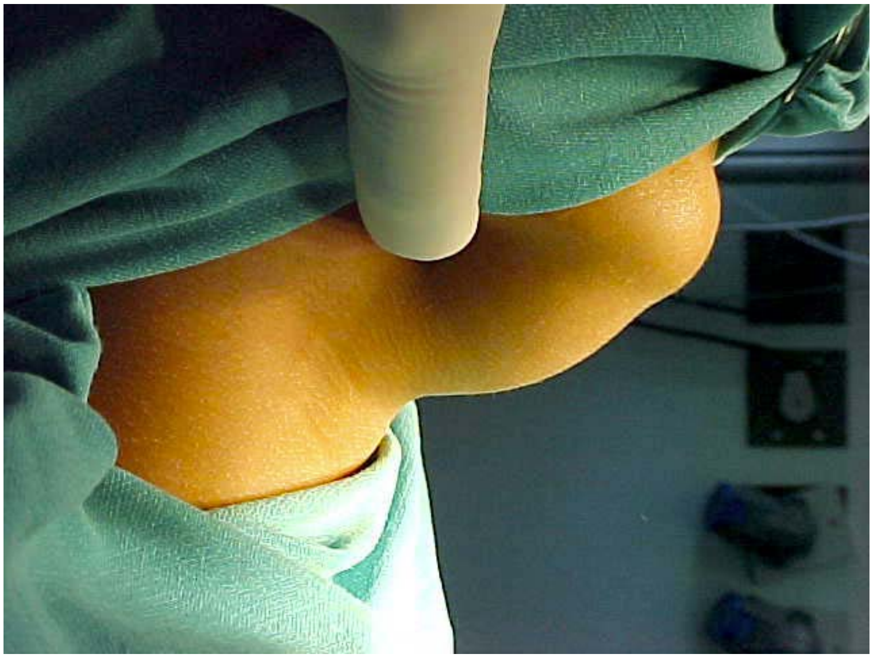

**FIGURE 1**. Preoperative view of the submental area showing a compressible mass in a 12-year-old child.

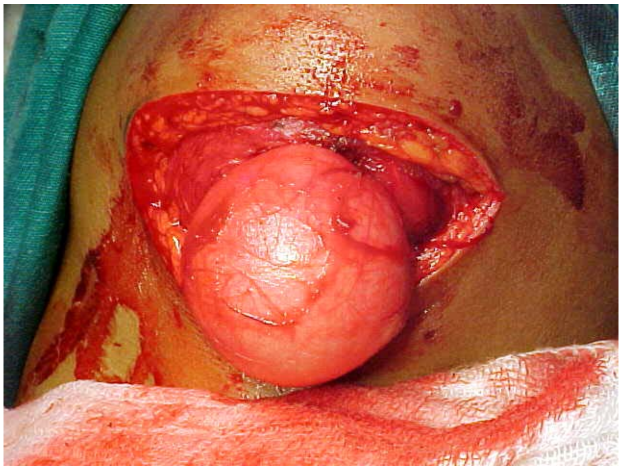

**FIGURE** 2. Transoperative view of the lesion after dissection.

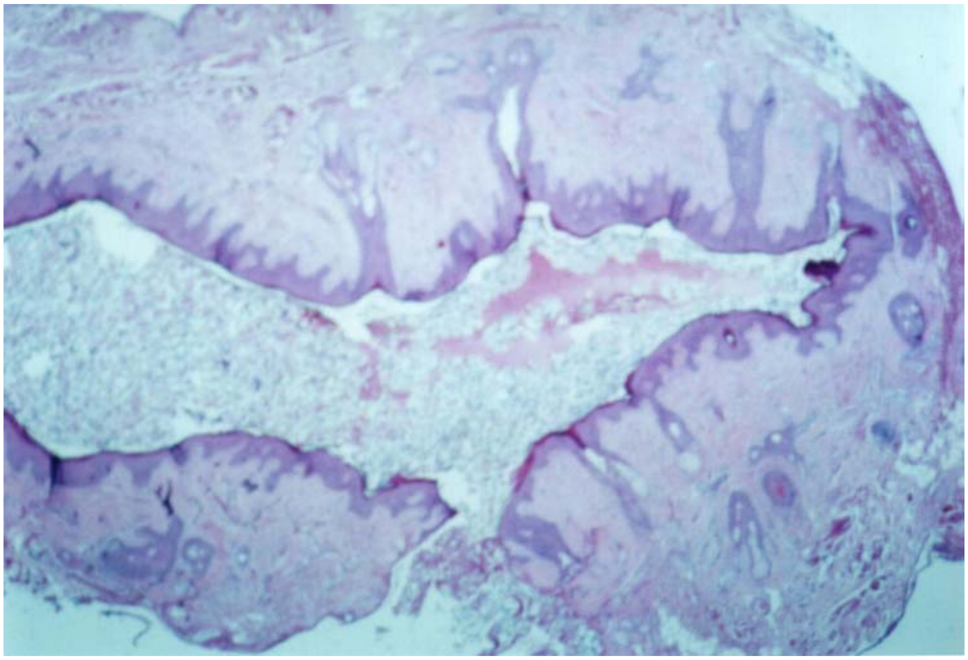

**FIGURE 3.** Low-power microscopy. The cystic lesion is lined by a stratified squamous epithelium. The lumen cyst is filled with a mixture of sebaceous and keratinized materials. Cyst capsule is composed of a narrow zone of compressed connective tissue with some dermal appendages. (H&E, original magnification X 25).

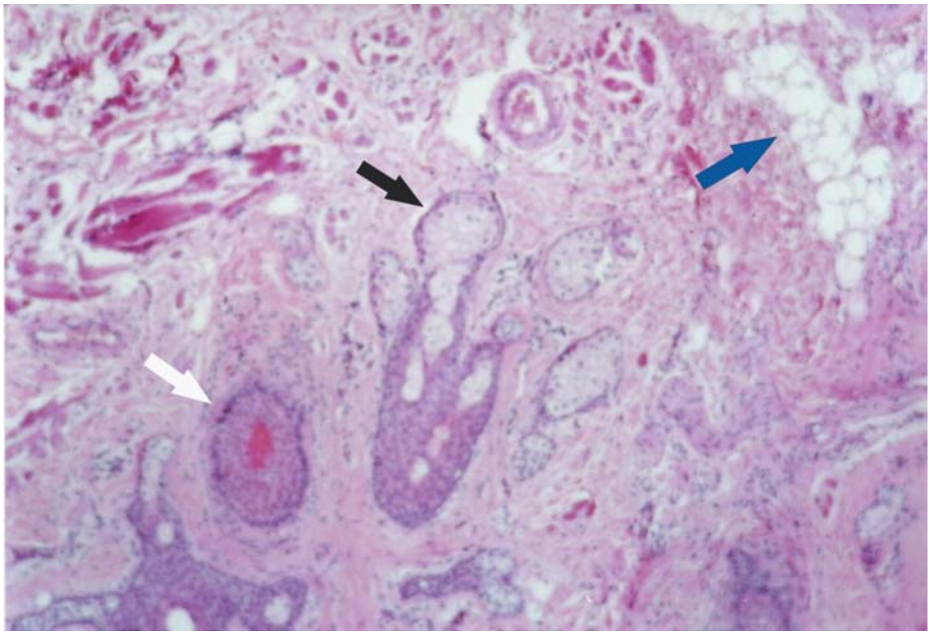

**FIGURE 4**. Medium-power microscopy. Capsule cystic exhibiting a dense fibrous connective tissue with variables numbers of dermal appendages including hair follicles (white arrow), sebaceous glands (black arrow), and adipose tissue (blue arrow). (H&E, original magnification X 40).

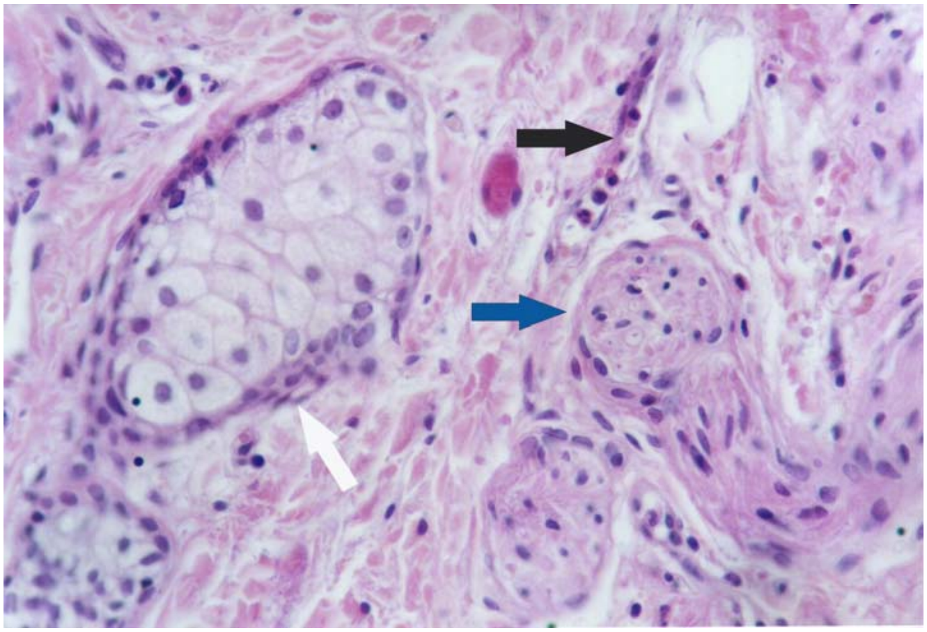

**FIGURE 5**. High-power microscopy. Details of cystic capsule exhibiting sebaceous glands (white arrow), nerve bundle (blue arrow), and blood vessels (black arrow). (H&E, original magnification X 100).

dermal appendages hair follicles, sebaceous glands, nerve bundle, and associated erector pili muscles (Fig 3). Connective tissue is generally free of inflammation. Diagnosis was dermoid cyst.

## DISCUSSION

Dermoid cysts are rare lesions in the oral and maxillofacial area. Mouth dermoid cysts account for only 0.01% of all oral cysts, and 1.6 % of all dermoid cysts[10]. New and Frich[11] related an incidence of 7% of this lesion between all head and neck cysts. They have been identified in patients in all ages, but they are found more frequently between ages 15 to 35[3,6,11]. According to Wallstad et al.[12], there is no sex predilection, and the cyst typically becomes evident in the second or third decade of life. This is in accordance to many others studies[3,5,6,11].

A dermoid cyst is defined as any cyst filled with sebumlike material and with evidence of specialized skin derivates from defective embryologic development (dysodontogenic)[3,5]. The most popular theory about its origin suggests that midline ectodermic tissue is entrapped during fusion of the mandibular (first) and hyoid (second) branchial arches in the third and fourth weeks *in utero*[3,6]. A second theory refers to dermoid cysts as acquired, post-traumatic, or implantation *in utero*, in which epithelial cells are forced into deep tissues[13,14]. In 1987, another study raised the possibility that midline dermoid or epidermoid cysts of the neck may represent a variant form of thyroglossal duct cysts in which the ectodermic elements predominate[15].

Clinically, these lesions present as slow-growing and painless swellings, soft, well encapsulated, and without lynphadenopathy[7], as in the present case. There was no presence of infection. Sinus tracts are not commonly associated with dermoid or epidermoid cysts[16,17]. Some studies have tried aspirations as a diagnostic device[1,18], others to provide better ventilation for the patient[19]. Any complications after aspiration were reported in the literature. Difficulty in speaking, breathing, and eating are normal complaints in patients presenting dermoid cyst in the floor of the mouth.

As an additional exam, ultrasonography (US) was performed in the present case. Ultrasound does not have deleterious defects[7], is inexpensive, rapid, and painless. It is efficient in diagnosing solid, vascular, or cystic lesions, as well as giving clear margins and relations of the lesion with adjacent structures[20]. Furthermore, it is important to establish the best surgical approach for transoperative visualization and to control the bleeding[21]. Other imaging devices are Computed Tomography and Magnetic Resonance Imaging. They are very useful techniques for diagnosing lesions, but are more expensive than US.

The differential diagnosis of these dysodontogenic cysts are an infectious process, plunging ranula, lymphoephitelial cyst, thyroglossal cyst, dermoid cyst, pleomorphic adenoma, and cystic hygroma. Other remotes possibilities are lipoma, neurofibroma, hemangioma, and lymphangioma [12].

There have been two different classifications for dysodontogenic cysts, histological and anatomical classifications. The histological classification is made according to the contents of the cyst lining. If only epidermis is present in the cyst lining, the lesion is called epidermoid. If skin annexes are present, the cyst is called as true dermoid. When tissues derived from the three germinal layers are present, it is called teratoma[3]. The anatomical classification is based on its relationship with geniohyoid, mylohyoid, and genioglossal muscles. This relation determines whether the lesions are in the sublingual, submental, or submandibular spaces[7].

The surgical approach will depend on its location. According to King et al.[7], 70% of 195 cases reviewed were excised by intraoral approach. Submandibular and submentual cysts are better enucleated by extraoral incision. Large cysts are enucleated by an extraoral approach, because it allows better visualization of the surrounding structures, bleeding control, and avoids intraoral contamination of the surgical wound[22]. When the cyst is located in the sublingual space, an intraoral incision is the first choice[23,24].

Recurrence rates are very low, and only four cases exist in which it is reported[22,25,26]. Other authors affirm that dermoid cysts do not recur if complete excision of the cyst is made[1,2,23]. When completely enucleated, using the right approach and good surgical technique, the possibility of recurrence is remote.

## CONCLUSION

Dermoid cysts are dysodontogenic lesions that are rare in the floor of the mouth. The clinical signs are very suggestive and additional exams are almost always necessary for diagnosing it, as for planning the surgical approach. The surgical approach is made according to the location of the lesion in facial spaces and recurrence after total enucleation is very rare.
